# PARPs database: A LIMS systems for protein-protein interaction data mining or laboratory information management system

**DOI:** 10.1186/1471-2105-8-483

**Published:** 2007-12-19

**Authors:** Arnaud Droit, Joanna M Hunter, Michèle Rouleau, Chantal Ethier, Aude Picard-Cloutier, David Bourgais, Guy G Poirier

**Affiliations:** 1 Health and Environment Unit, Laval University Medical research Center, CHUQ, Québec, Canada; 2 Eastern Quebec Proteomic Center, CHUQ, Quebec, Canada; 3 Caprion Pharmaceuticals, Montréal, Canada

## Abstract

**Background:**

In the "post-genome" era, mass spectrometry (MS) has become an important method for the analysis of proteins and the rapid advancement of this technique, in combination with other proteomics methods, results in an increasing amount of proteome data. This data must be archived and analysed using specialized bioinformatics tools.

**Description:**

We herein describe "PARPs database," a data analysis and management pipeline for liquid chromatography tandem mass spectrometry (LC-MS/MS) proteomics. PARPs database is a web-based tool whose features include experiment annotation, protein database searching, protein sequence management, as well as data-mining of the peptides and proteins identified.

**Conclusion:**

Using this pipeline, we have successfully identified several interactions of biological significance between PARP-1 and other proteins, namely RFC-1, 2, 3, 4 and 5.

## Background

Proteomics aims to identify, characterize and quantify all of the proteins expressed by a given living organism, tissue or cell line[1]. Typically, this approach subjects protein mixtures to proteolytic digestion prior to liquid chromatographic separation and MS/MS analysis of the resulting peptides [2]. Several database search engines, notably Mascot[3], Sequest [4], and X!Tandem [5] assign probable peptide sequences to MS/MS spectra and infer the identity of the proteins present in the sample analyzed. High-throughput proteomic experiments generate large data sets of protein identifications, which can only be properly validated and reported through adequate data processing [1,6,7]. Subsequent integration, sorting and comparison of these datasets pose significant challenges, especially when simultaneously analysing multiple experiments.

One of the most effective approaches to elucidate the biological function of a protein is the identification of its interaction partners. We are only now beginning to appreciate the nature and complexity of networks of interacting proteins. The unravelling of any such network using traditional biochemical approaches remains a significant challenge. Recently, however the application of high-throughput technologies, such as large-scale yeast two-hybrid analysis and mass spectrometry coupled to immuno- or affinity-based capture has made possible the rapid generation of huge protein interaction datasets [8-10]. As consequence, researchers often face the dilemma of how to effectively utilize all available data. Investigators relying solely on a traditional approach to draw conclusions or set research priorities are likely to find themselves outpaced by peers who combine *in silico *biology and empirical methods. Thus for protein interaction studies, there is clearly a need to develop a systematic and stepwise *in silico *approach to predict potential interactors. This approach will most likely improve our understanding of how complex biological systems work.

The need to develop a Laboratory Information Management System (LIMS) for researchers in the field of proteomics that would allow to track, archive and aid in a greater understanding of how biological systems work has been recognized. In 2002, Cho and co-workers developed an original LIMS for proteome research (YPRC-PDB)[11], constructed using a commercial database (RDB). They intended to establish YPRC-RDB as a proteome data warehouse. In 2003, Goh and co-workers [12] developed SPINE2, a LIMS for structural proteomics, constructed with MySQL and Perl, and also designed to work as a pipeline to public data resources. In 2004, Garwood and co-workers [13] developed PEDRo, The Proteomic Experimental Data Repository, constructed with a native XML database, Xindice with an ambitious Apache Software Foundation basis. The XML-based document format has been chosen for communication that the other formats. The native XML database has great potential, but many have critical limitations for proteomic research. On the other hand, commercially available LIMSes (Amersham Biosciences and Bio-Rad Laboratories Inc, etc.) have also been developed and released, but they are not exactly suitable for laboratories like ours because the generic solutions are first and foremost prohibitively expensive. These systems are usually re-packagings of applications developed for the pharmaceutical industry for drug discovery and development.

The focus of our laboratory is the study of the action of poly(ADP-ribose) polymerases (PARPs) and their role in the cell. Poly(ADP-ribosylation) is a post-translational protein modification consisting of long chains of poly(ADP-ribose) (pADPr) synthesized by PARPs at the expenses of NAD+. Poly(ADP-ribose) chains are short-lived owing to the activity of the poly(ADP-ribose) glycohydrolase enzyme, which catabolizes the pADPr within minutes of its synthesis[14]. The PARP family may comprise as many as 17 members which share a common catalytic domain responsible for the synthesis of poly(ADP-ribose) [15-17]. The best characterized and abundant member of this family is PARP-1, a 113-kDa nuclear protein comprising a DNA-binding domain made of two zinc fingers that allow PARP-1 to be rapidly activated in response to DNA damage. Poly(ADP-ribose) crucially contributes to chromatin remodelling, DNA damage repair, regulation of transcription, and cell division [18-20]; and PARP-1 is an important actor in many key cellular processes, including BER, transcription, and apoptosis.

We herein describe the architecture and major features of a web-based utility called "PARPs database" (PARPs-DB), which is designed to rationally organize the protein and peptide data generated by the LC-MS/MS analysis of tryptic digest of proteins that co-immunoprecipitate with PARPs proteins into reports meaningful to biological researchers. PARPs-DB offers a LIMS work environment to annotate and study protein-protein interactions and its easy-to-use relational data management system can rapidly supply information pertaining to the biological characteristics of a majority of proteins in a proteomic dataset. The major features of our LIMS are flexibility, compactness, and connectivity to public databases.

Also presented is a list of previously unidentified PARP-1 interactors that were found via affinity co-immunoprecipitation, as well as the analysis of these new PARP-1 interactions generated through PARPs-DB.

Given the advantages provided by an *in silico *approach that can predict or prioritize potential interactors, it seems reasonable to propose that PARPs-DB will become an essential tool for initially evaluating novel hypotheses and will offer improved rationale for the prioritization of potential interactors. In effect, by facilitating the processing of protein-protein interactions and the selection of the most promising interactors (to be submitted first to empirical measurements) PARPs-DB should lower the cost and shorten the time necessary to discover the most biologically significant interactions between PARPs and other proteins.

We don't have infrastructure to access on-line but the PARPs database source codes and user documentation are available for the scientific community [21]. Tools from the Sashimi project that were used in PARPs-DB are also available [22]. Licensed programs in Sourceforge such as Mascot, Sequest or Oracle were not included in the PARPs repository. Dialects for MySQL and PostgreSQL servers were developed in alpha version and are available upon request. Further information on these scripts can be obtained from the corresponding author (see Additional file 1).

## Construction and Content

### Design of the PARPs database software

PARPs Database consists of a core system of services that provide underlying system functionality. Modules, which provide most data handling and analytical support (such as LC-MS/MS data mining), plug into the core. This design means the platform is easily expandable: the architecture allows new analytical modules to be added and integrated without having to modify the core system. PARPs database was designed to be platform-independent and easy to maintain, and is implemented in Solaris Sun Operating system 10 (Sun Microsystems, Santa Clara, CA, USA). It requires access to an Oracle 10 g relational database (Oracle, Redwood Shores, CA, USA), with which it communicates through an abstraction layer that isolates the core system from subtle differences between Oracle database builds. The user interface supports the use of the Apache server[23] for external access via the Hyper Text Transfer Protocol (HTTP). It consists of a set of programs, written in the Practical Extraction and Report Language (Perl) and in PL/SQL, which generates the user interface in Hypertext Transfer Markup Language (HTML), using Cascading Style Sheets (CSS), eXtensible Markup Language (XML) Style sheet Language Transformation (XSLT), and the Scalable Vector Graphics language (SVG). A web browser is used as the user interface of the LIMS, because it is universally available on most client systems. Internet Explorer 6.0 or later, Netscape 7.1 or later, or Firefox version 1.03 or later should be installed in the client PC.

Dialects for PostgreSQL and MySQL servers were implemented, and support for Microsoft SQL is under development.

### Database design

**Figure 1:**
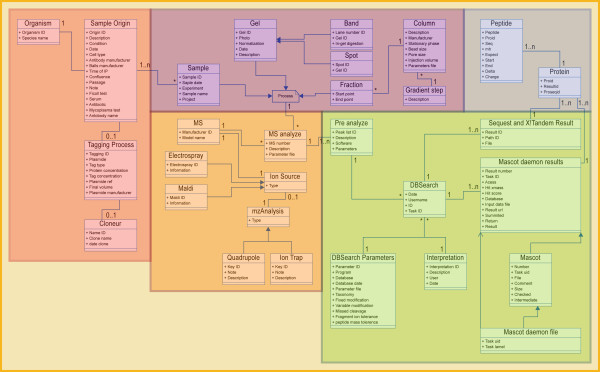
**A simplified schematic representation of the structure of PARPs database**. Different classes (rectangles) with their associations (lines) are shown. A class is described by its attributes, e.g. a sample can be specified by its name, date. Colours denote sample generation (red), sample processing (purple), mass spectrometry (orange), MS results analysis (green), and in silico MS results analysis (blue). The full-scale schema is available on-line as supplemental Figure.

Figure 1 outlines the database schema for the data pertaining to experimental protocols, data analysis and results (the full-scale schema is available on-line as Supplemental Figure 1). The database is defined in the Unified Modeling Language [24], which is a standard notation designed to improve the process of developing large software systems [25]. In this context, it allows us to describe experimental methods, results, and subsequent analyses in an implementation-independent manner. UML schemas (Figure 1) are referred to as class diagrams. They consist of boxes (classes), representing important entity types, connected by various types of lines and arrows signifying the relationship between them.

**The sample origin (shown in red) **holds basic information, such as the specific biological material used, which subcellular fraction was studied, and the experimental conditions to which the organism was subjected. Sample origin has also two offsprings: 'organism' holds the name of the species/strain used and a list of the relevant gene/mutations carried; and 'tagging process' describes the labelling of the parts of a combined sample for differential expression studies, such as isotope-coded affinity tag (ICAT) mass spectrometry[26].

**The sample (shown in purple) **simply holds an identification code (laboratory-specific), the production date, and the name of the responsible person.

The next classes represent sample's processing step before moving on to run a mass spectrometry experiment. For example, running a two-dimensional gel with sample, then putting a spot from that gel through two-dimensional liquid chromatography. The class HPLC describes the equipment's origin, its dimensions, the stationary phase, the pore size in the beads, the total injection volume, and the flow rate. The class gel capture the description of the gel, the image analysis software used, and whatever images of the gel are available, referred to by URIs (Universal Resource Identifiers). There are also several parameters describing the gel itself (for example, percent acrylamide in the mix, the solubilisation buffer and stain used, a measure of the total protein on the gel, the in-gel digest).

**Mass spectrometry (shown in orange)**. Details about the makeup of the mass spectrometry machine is stored in seven classes. Source is an abstract class that will, in practice, be either MALDI or Electrospray, each of which has its own set of fields (voltages of various kinds; tip, solvent, and interface details for electrospray; laser wavelength and matrix type for MALDI runs). Instrument represents the mass analysing and fragmentation section of the mass spectrometer (for example, Quadrupole, Ion Trap, or Collision Cell, each with its own parameters).

**MS results analysis (shown in green). **To perform a protein identification, a particular Peak list would be submitted to an identification tool, such as Mascot, Sequest and X!Tandem. The classes 'DBSearch Parameters' capture information about who processed data, when they did it, what program they used, what database was used, what errors were taken into account when searching, what potential modifications were allowed on proteins from the sample that generated the peak list.

**The protein tables (shown in blue) **store identifiers (accession numbers) that point to external web-based information sources. Short text annotations such as Gene Ontology [27] descriptions, descriptions of functional or structural regions within the protein sequence, and information about associated diseases and biological pathways are also stored when available. While the identifiers serve as links to external databases and web pages, the annotations stored within PARPs-DB are human readable and easily searchable. PARPs-DB also supports input of local protein sequences and annotations, as well as pointers to local databases. A sequence or annotation marked as "defunct" will not automatically be deleted from the database, which means old FASTA files can be reanalysed with new annotations even if their records have been deleted or replaced by subsequent information in the primary source.

The database was designed to contain a minimal amount of information but still sufficient data to allow effective Structured Query Language (SQL) queries. These queries enable ready access to any information stored in the database as well as in the XML files generated by the data analysis server. With the tables and XML files serving as the primary data storage objects, the relational dataset is relatively easy to build, maintain and query.

### LC-MS/MS data analytical module

A key design element of PARPs database is the ability to generate analytic modules that plug into and use the core of PARPs system. Three pivotal LC-MS/MS tools integrated in PARPs-DB are the peptide-spectrum matching programs: Sequest, X!Tandem and Mascot. We have also included PeptideProphet to validate peptides assigned to MS/MS spectra [28] and ProteinProphet to infer the proteins[29] present in the sample from the list of observed peptides. These open-source tools are components of the Trans Proteomic Pipeline (TPP) from Seattle Institute for Systems Biology (ISB) [30]. To access MS/MS data, RAW files were converted to the open file format (mzXML or mzData) using Readw.exe from Sashimi for LTQ mass spectrometer for example or our own conversion software. Sashimi [22]is a project initiated at the ISB that aims at providing the scientific community with free and open-source software tools for the downstream analysis of mass spectrometric data. Sashimi is focused on the bioinformatics standards necessary to the set up of a generic proteomic pipeline using common output formats at each processing step. We have also integrated three executable: Sequest2XML, Mascot2XML and Tandem2XML, also from Sashimi, to convert search engine outputs (DAT, OUT and XML files) to pepXML[31].

### Links to public databases

The underlying protein knowledge base used by PARPs-DB was extracted from multiple online resources, based on cross-references. Five human gene and protein data sources were integrated within PARPs-DB : protein databases maintained by IPI [32] and UniProt [33], and three NCBI databases: Entrez Gene [34], RefSeq [35], and GenPept. Three protein-protein interactions databases were also included in PARPs-DB's knowledge base : the Biomolecular Interaction Network Database (BIND)[36], the Database of Interacting Proteins (DIP)[37] and Human Protein Reference Database (HPRD) [38]. For each identified protein stored in PARPs-DB, the data analysis server gathers the protein's function, sequence and post-translational modifications from the above sources and presents the extracted data along with the identified protein. Different strategies have been used to update our databases. For protein databases such as Uniprot and IPI, we use Perl scripts to download Fasta files from the Uniprot and EBI server. A report of the new release updates is produced. For protein-protein interaction databases such as HPRD and BIND, monthly updates are also performed through Perl scripts.

### Protein-Protein interaction viewer

Finally, in order to visualize protein-protein interaction networks, we have developed a protein-protein interaction viewer, in Java language (Java JDK 1.4.2_05 and Netbeans 3.6). This viewer uses three libraries: Xerces Java Parser 2.6.2, Piccolo Java 1.1, and JDOM 1.0 this last library being used to manipulate and parse the XML files. The central organization of the protein-protein interaction viewer is a network graph with molecular species represented as nodes and intermolecular interactions represented as links, that is, edges between nodes. This application provides basic functionality for integrating data on the graph, a visual representation of the graph and integrated data. Data are integrated with the graph model using attributes. Graphical browsers allow the user to examine all attributes on the currently selected nodes and edges.

One of the most fundamental tools for interpreting molecular interaction data is visualization of nodes and edge as two dimensional network. It utilizes a relaxation layout algorithm which attempts to prevent overlapping of nodes. This viewer is small, stable, multi-platform and simple to use. It can function as a stand-alone applet or be integrated into a web application.

### PARP-1 co-immunoprecipitation

#### Cell culture

Human cervical carcinoma HeLa cells obtained from ATCC (Manassas, VA, USA) were cultured in Dulbecco's modified Eagle's medium (DMEM) supplemented with 10% fetal bovine serum, 2 mM L-glutamine, 100 U/ml penicillin and 100 μg/ml streptomycin in an humidified atmosphere of 5% CO_2 _at 37°C. All the above-mentioned reagents were purchased from Invitrogen (Burlington, ON, Canada).

#### Immunoprecipitation of endogeneous PARP-1

Cells grown in 150 mm culture dishes were washed with ice-cold phosphate-buffered saline (PBS). 400 μl/dish of ice-cold lysis buffer (175 mM KPO_4_, pH 8.0, 150 mM NaCl, 1% NP-40, 1 mM DTT, 0.5 mM PMSF and Complete™ protease-inhibitor cocktail (according to Roche diagnostics instructions)) was added to the cells. Cells were harvested using a cell scraper. Lysed cells coming from three dishes were pooled then gently mixed by inversion for 1 hour at 4°C and centrifuged 10 minutes at 6000 g at 4°C to remove insoluble cellular debris. The cellular extract was mixed with 180 μl of magnetic beads coupled to protein G (Dynal, Invitrogen) and 8 μl of monoclonal antibody to PARP-1(F1-23)or 8 μl of normal mouse IgG as control and incubated during 2 hours at 4°C with rotation. The beads had been previously blocked during 1 hour with 1% BSA and washed with lysis buffer. At the end of the incubation period, the beads were washed 3 times with lysis buffer. 180 μl of 2× Laemmli SDS sample buffer containing 5% (v/v) β-mercaptoethanol was added to the beads and they were placed in a boiling bath for 5 minutes to elute the immunoprecipitated proteins.

#### Protein separation and digestion

Immunoprecipitated proteins were separated by SDS 8% PAGE. The gel was fixed for 30 min with 10% (v/v) methanol and 7% (v/v) acetic acid solution, then stained with SYPRO Ruby fluorescent protein stain (Bio-Rad, Hercules, CA, USA) according to the manufacturer's instruction. The entire protein profile of the immunoprecipitated proteins was sliced from the gel into 50 bands using a gel excision Lanepicker™ (The Gel Company) and placed into a 96-well plate. In-gel protein digests were performed on a MassPrep™ liquid handling station (Waters) using sequencing-grade modified trypsin (Promega) according to the manufacturer's instructions. Peptide extracts were evaporated to dryness using a SpeedVac™ and resuspended in 10 μl of 0.1% formic acid in water.

#### LC-MS/MS

Final extracts were analysed by LC-MS/MS using an LCQ-DECA XP mass spectrometer equipped with a nanospray ESI (electrospray ionization) source and a Surveyor autosampler and HPLC system (Thermo Electron). A 5 μl volume of extract was first focused on a Peptide CapTrap™ (Michrom Bioresources) and then loaded on a Biobasic C_18 _PicoFRIT™capillary column (PFC7515-BI-10; New Objective). Elution of peptides was performed using a linear acetonitrile gradient (0–60%) over 20 min at a flow rate of approximately 200 nl/min (buffer A: 0.1% formic acid in water; buffer B: 0.1% formic acid in acetonitrile). MS, including collision-induced dissociation, was performed in an automated fashion using the dynamic exclusion option.

#### Protein identification

Peptides were assigned MS/MS spectra by searching using Sequest (version 2.0 SR2), Mascot (version 2.1) and X!Tandem (2006.04.01.2) and the assignments were also validated with Scaffold software (Proteome Software Inc.; version Scaffold-01_03_02). MS/MS spectra were searched against the IPI human protein database (version 3.01)[32] to which the sequences of protein constructs, proteins of interest, and common contaminants were added. Searches were performed specifying complete (fixed) carbamidomethylation modification of cysteine (+57 Da) and oxidation of methionine (+16 Da) residues. The digestion enzyme parameter was set to trypsin. The proteins identified in this paper were obtained with a Scaffold probability cut-off of 80%.

#### Western blots

Total protein extracts and proteins eluted from the immunoprecipitations were separated by 8% SDS-PAGE and then transferred onto a 0.45 μm pore-size PVDF membrane (Millipore, Bedford, MA, USA). After incubating 1 hour with the blocking solution (PBS with 0.1% (v/v) Tween-20 (PBS-T) containing 5% non-fat milk), the membrane was probed with primary antibodies to PARP1 (C2-10, mouse monoclonal 1:5000) or RFC1 (Replication factor C, 140 kDa subunit, rabbit polyclonal antibody 1:2500) (Bethyl Laboratories, Montgomery, TX, USA) overnight at room temperature with shaking. After washing with PBS-T, species-specific horseradish peroxidase-conjugated secondary antibody was added for 1 hour at room temperature. The signals were finally detected with the Western Lightning™ Chemiluminescence reagent plus kit (Perkin Elmer, Boston, MA, USA).

## Utility and Discussion

### Protein interaction research workflow

**Figure 2:**
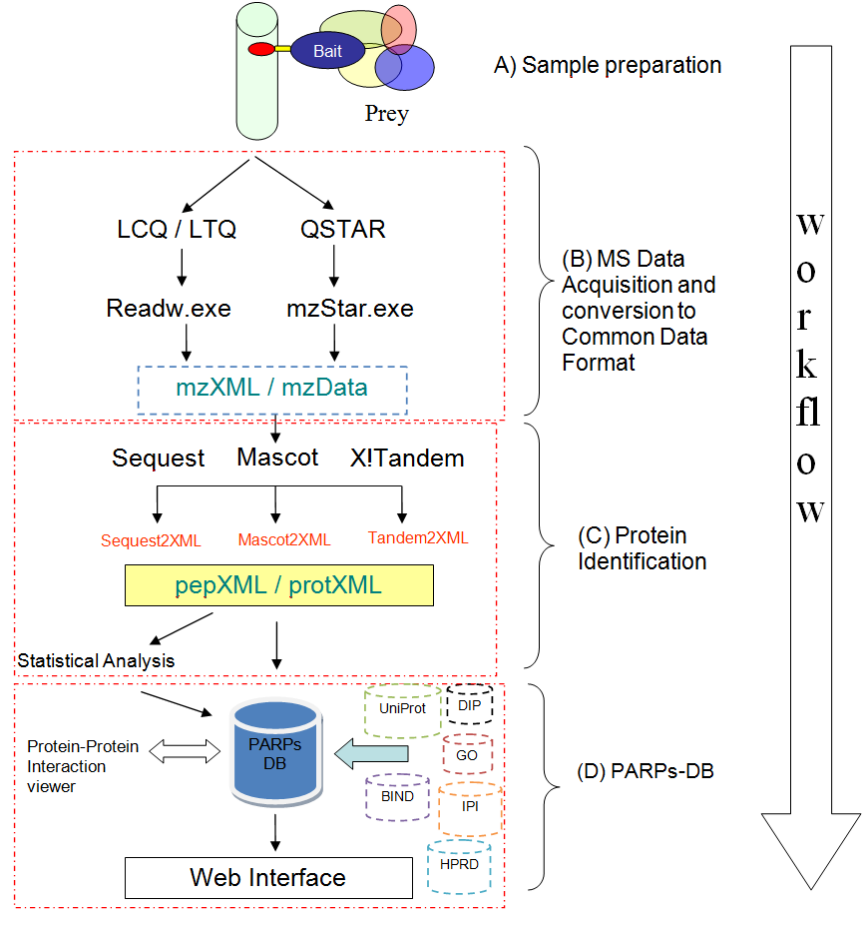
**Schematic overview of the approach for PARPs protein interaction research**. The steps are (A) PARPs co-immunoprecipitation and with interactors; (B) generation of mass spectral data; (C) peptide sequence assignments using different search engines and protein identifications using different methods of inference; (D) the annotations and results are loaded automatically into PARPs database for viewing, annotation and analyses.

The workflow of the PARPs protein interaction research is illustrated in Figure 2. In our LIMS, the data processing is divided into sections corresponding to the four main steps: sample preparation, MS Data acquisition, protein identification, and PARPs-DB.

*The sample preparation *(Figure 2A) section allows laboratories to track and organize biological experiments and view the workflow of those experiments.

For the purpose of *MS data acquisition *(Figure 2B), different types of mass spectrometers, using different methods for ionization and mass determination, may be used. As the instruments from diverse suppliers use different formats to store instrument parameters and spectral data, PARPs-DB uses parsers to convert the data from the different mass spectrometers (*LTQ and QSTAR) into mzXML/mzData. PARPs*-DB is very flexible. Additional mass spectrometers and converters to XML files can be easily included. mzXML and mzData are designed to encompass all of the information required by the peptide-spectrum matching software such as Sequest and Mascot. Moreover these data representations, developed respectively by Seattle Institute for Systems Biology (ISB) and the European Bioinformatics Institute (EBI), provide an OS and architecture-independent standardized file format and remove the burden of having to support multiple native formats. The Sashimi project currently provides converters from native binary files to mzXML (example ReAdW, convert the RAW files generated by Xcalibur). Unfortunately, there is no program available to convert proprietary binary format to mzData. Therefore, the easiest way to publish a peak list data in mzData today is to convert mzXML into mzData using any XML parser.

By converting all native binary data to mzXML/mzData and using these standards at the start of our analysis pipeline, the downstream software tools, specifically the database search module and raw spectral viewer, can be used in a uniform manner regardless of the instrument used to acquire the MS/MS spectra.

In order to identify proteins from the tandem mass (MS/MS) spectra, *the protein identification section *(Figure 2C) is used to submit the spectra to three search engines, namely Sequest, Mascot and X!Tandem. PARPs-DB MS/MS analytical module stores, shares, analyses, mines and publishes tandem MS data. This module supports pepXML, which stores the results of peptide sequence assignments and subsequent peptide-level analyses in a XML files. After search results have been written or converted to pepXML, they can uniformly be subjected to peptide-level applications and viewed without regard to the algorithm used to assign peptides to MS/MS spectra. Users can examine individual LC-MS/MS runs and groups of runs using complex customizable analytical filters for peptides and proteins on the various search engine specific scores (XCorr for Sequest, log(e) for X!Tandem). These filters can be saved for later use. Finally, protein identifications are stored in protXML. The multiple possibility discordant sequence identification presented in each run is encompassed by protein ProteinProphet which all peptide evidence is combined. This data in XML format (developed by Sashimi) stores protein identifications inferred from input lists of peptides and their subsequent protein-level analyses. After protein identifications are converted to protXML, protein-level analyses such as protein quantification can proceed and results viewed without regard to the method used to infer protein identification. With the help of this standard, we have used a set of open source tools, PeptideProphet and ProteinProphet, which provide a standardized method of validating MS/MS data. For example, accurate probabilities provided by PeptideProphet and ProteinProphet serve as guides for the interpretation of peptide and protein identifications, respectively, and enable the prediction of false positive error rates that can be used as objective criteria for the comparison of data sets generated by different researchers. This module interacts with the protein annotation (described in the next section) module to display information rich annotations for putative protein identifications.

This last section represents the *PARPs database *(Figure 2D). Following the execution of the data processing methods described above, the results are loaded automatically into PARPs-DB for viewing. The database system is interconnected with the protein annotation module (Figure 2D). This module manages protein sequence annotations to help investigators cope with any newly updated or revised information about proteins and their properties. Sequence annotations are automatically updated. However, updates to the system are stored incrementally so that any previous version of a database annotation can be retrieved at any time. Protein annotations interact closely with the protein identification section to allow users to view up-to-date descriptions of protein sequence that have been identified.

A sequence or annotation marked as "defunct" will not automatically be deleted from the database, which means old FASTA files can be reanalyzed with new annotations even if their records have been deleted or replaced by subsequent information in the primary source. Specific databases such as UniProt, IPI, RefSeq, BIND, HPRD, Gene Ontology are downloaded in the PARPs-DB.

### Accessing and navigating experiments in PARPs database

**Figure 3:**
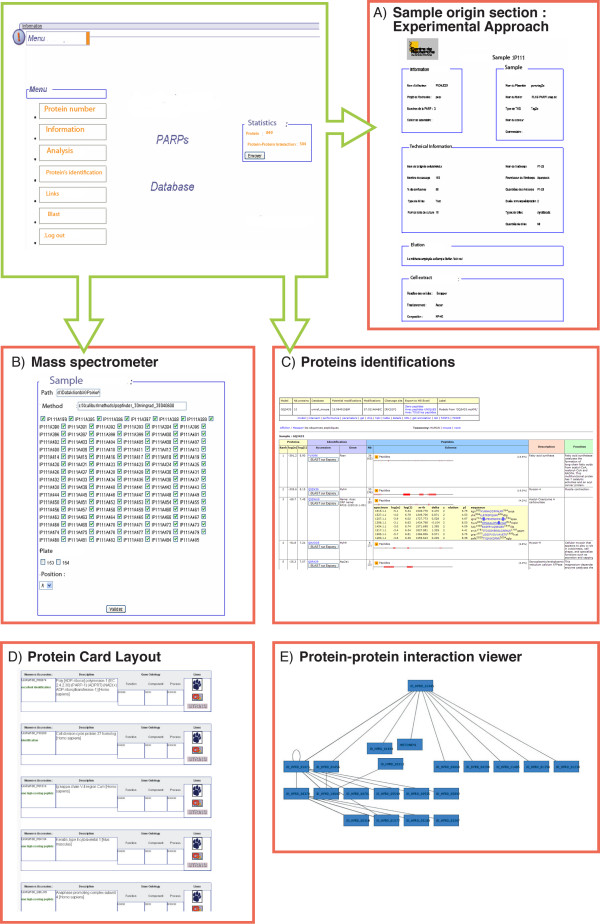
**PARPs database user interfaces**. (A) In the Sample Origin section the user can enter experimental parameters and visualize the experimental protocol; (B) In the Mass Spectrometry section the user defines the parameters of the mass spectrometer; IPxxx is the identification of immunoprecipitation experience. (C) The Protein Identification section summarizes protein identifications, including protein accession number, entrez gene accession, number of peptides identified and a protein summary function; (D) The Protein Card Layout contains links to a variety of external public sources; (E) The Protein-Protein Interaction Viewer allows the user to display protein-protein interactions from internal and external (publicly available) data sources. The full-scale schema is available on-line as supplemental Figure.

To facilitate data analysis, a graphical user interface (GUI) was developed. The GUI guides the user through all steps of the experiment to enter information such as immunoprecipitation methods, gel images, mass spectra, search engine results, etc. (Figure 3), which ensure a complete documentation of the experimental setting. After all necessary data have been stored in the system, the user can select data sets for visualization.

To access the LIMS server using a web-based client, the user must first login with an authorized username and a given password. PARPs-DB users are authenticated against a **Lightweight Directory Access Protocol **(LDAP) provider such as institution's name server. Experimental data and other materials are stored in projects and their sub-folders, much like a file system. Each project has one or more groups of users associated with it, and each group can have a distinct set of permissions (e.g., read only, read and write) to each of the project's folder. When users log in, the authorization system determines what data they have permission to view, edit, and/or delete and provides access accordingly.

Inside the PARPs-DB, there are three main sections, "*sample origin*," "*mass spectrometry*" and "*sample results*" corresponding to different steps of a proteomic experiment. These sections allow users to store experimental parameters, results and annotations. Each section has a distinct set of permissions. For example, a molecular biologist cannot access the mass spectrometry section, and conversely, mass spectrometer users cannot access the molecular biology section. In each section, we have developed tools to help the user reduce the time needed to analyse data.

First, the *sample origin *section enables the user to enter experimental parameters by selecting a number of options. Experimental information includes cell type and cellular conditions, method of gene transfer (when applicable) and gene sequence, and details of the immunoprecipitation method such as lysis buffer composition, antibodies, cell lysis. The user can print experimental details entered in the database (Figure 3A). For example, an image of a stained gel showing proteins immunoprecipitated in the described experiment may be loaded into the database.

The second section of PARPs-DB is the *mass spectrometry section*. In this section, the user may define the parameters of a mass spectrometry experiment including: the plate number, the spot position, method files (Figure 3B) and parameters for search engines. A tabular file is generated to upload the list of samples into the mass spectrometer software. At the end of MS/MS analysis, the raw data is transformed in mzXML and mzData automatically in the background. Unfortunately, all converters run on windows environment because some Windows specific libraries are necessary. After each run, the binary data files are transformed locally in XML files on windows computer. After the conversion, each XML files are transferred with Secure File Transfert Protocol (SFTP) to UNIX server.

The user accesses database searching through another section of the user interface in order to set specific search engine parameters such as the database to be searched and amino acid modifications. The data pipeline will submit the mzXML or mzData to the search algorithms and manage the specification of search parameters and FASTA files. Once analysed, the system offers graphical and tabular views of the experimental steps and their input and output. Users can monitor the progress of their searches via the web interface.

The last section of PARPs-DB is the *sample results *section. Access to LC-MS/MS results is available in this section, which shows protein and peptide identifications in the list view of the PARPs database. The data may be sorted according to certain experimental protocols (e.g. digestion) or according to the identification probabilities (Figure 3C). Display columns include the UniProt[33] or IPI[32] or RefSeq [35] annotation, the number of uniquely identified peptides per protein, and the total number of identified peptides per protein. MS/MS search results can be evaluated using this module, which allows proteins and peptides to be sorted and filtered by various criteria. Each identified protein is linked to the protein annotation module (see below), through which it is automatically linked following parsing of the FASTA file, allowing access to a variety of up-to-date external sources (Figure 3D). The accession numbers of proteins identified from the Sequest, X!Tandem, or Mascot searches are matched with those from IPI, and specific information regarding the protein of interest is automatically retrieved and displayed within the database window. Additional information from the software-assisted identification of the protein is displayed in a portal view, including identified peptides. The purpose of this feature of the PARPs database is to automatically connect protein identifications to their function and other relevant biological information extracted from external databases. A statistics module within PARPs-DB provides basic information about each experiment in the form of charts (e.g. gene ontology annotations, the number of peptides per protein, proteins identified with a certain ProteinProphet probability, etc.). In addition, the database allows for the comparison of data from different experiments at protein and peptide levels. Users are able to query the database, add notes to specific identifications, and select and export lists of interesting proteins including annotations.

Different tools are accessible throughout PARPs database navigation. These include BLAST, CLUSTALW and our protein-protein interaction viewer, a graphical tool that is linked to PARPs-DB. The viewer displays protein-protein interactions from the PARPs-DB (Figure 3E). The protein interaction network is shown as nodes (proteins) and edges (interactions). The interaction network can also be displayed with the annotations for the proteins in the nodes. Each node is linked to the protein annotation module. We displayed the confidence of each external protein-protein interaction using the thickness of the edge (default value 2). Redundant interactions independent reports in each external data source were assigned confidence values of 3. In addition, the colour (red: Bind database, blue: HPRD database, or green: PARPs database) of the edges can be selected to indicate the respective data sources.

The user can scan all the deposited internal (our protein-protein interaction assays) and external protein-protein interactions (from publicly available data sources: INTACT, BIND, HPRD, String) in the database. Information about protein-protein interactions beyond the target protein is shown in the interaction network to visually characterize the protein network. Proteins of interest can be searched by either accession number or keywords. When users input the accession number of a protein, the protein interaction network is shown as nodes (proteins) and edges (interactions). The interaction network can also be displayed with the annotations for the proteins in the nodes. Each node is linked to the protein annotation. Data and results can be exported to other formats including PSI-MI, Excel and DTA (Sequest files) for additional analysis using other tools. This method was created to exchange data easily between different laboratories.

### Using proteomics standards in PARPs database

A major obstacle to uniform proteomic analysis has been the great heterogeneity of data formats at three distinct levels: different mass spectrometers output their raw spectral data in different proprietary formats, methods that assign peptides to MS/MS spectra output their results in a variety of formats, and different methods to infer protein identifications from lists of peptides output their results in different formats. The proteomics community has recognized this problem and is tackling it through the formation of working groups (Proteomics Standard Initiative; Institute for Systems biology) concerned with the development of standards for the capture and sharing of proteomics data. The PARPs database was developed in agreement with the HUPO-PSI (Human Proteome Organization-Proteomics Standard Initiative), which includes PSI-MI (Molecular Interactions), MS (Mass spectrometry) and GPS (General Proteomics Standards). The GPS development of standard ways to represent proteomics data and an agreed minimum required level of detail are both urgently required to facilitate the analysis, dissemination and exchange of proteomics data. Minimal Information about Proteomic Experiment (MIAPE) is a proposed standard format for proteomics covering 2-DE and MS. The PARPs database contains classes derived from PEDRo[13]. As mentioned earlier, the latest proteomic standards such as mzXML, mzData and PSI-MI have been incorporated into our pipeline. We expect the new format such as mzXML to facilitate the exchange and publication of MS-based proteomics data and that our PARPs database will provide a consistent platform for the development of new analytical tools.

We have developed PARPs-DB as an in-house, flexible Laboratory Information Management System (LIMS) which integrates all aspects of the study of protein-protein interactions by mass spectrometry-based proteomics, from sample processing information to protein interactions visualization. PARPs-DB allows easy network access to public databases, manages the output of different search engines (MASCOT, X!Tandem and Sequest) and supports HUPO's Proteomic Standard Initiative formats. Although several LIMS have been made freely available during last few years, none of these existed when work on the PARPs database was initiated in 2002. Advantages of PARPs-DB over these free LIMS includes: integration of all data related to the mass spectrometry-based study of protein-protein interactions; multiple search engine support; and user-friendliness.

It is indicative of the poor availability of appropriate commercial systems that development of in-house LIMS such as PARPs-DB started at around the same time in different laboratories across the world, in order to fill the urgent need to automate proteomic data storage and analysis. Commercial LIMS now available remain however prohibitively expensive for small MS facilities and more rigid than an in-house system. These systems often include rigorously defined user roles and access privileges, as well as extensive auditing of data file changes. Although essential to pharmaceutical companies filing drug applications which must comply to regulatory standards (e.g. 21 CFR part 11), these features can hamper academic research because files can not easily be modified, maintained, and updated. Additionally, integration of the output of a new mass spectrometer can be more difficult and costly than with an in-house, flexible system. Finally, available LIMS from mass spectrometer vendors present many limitations: there is notably no integration of the data generated by other vendors' instruments, nor of the data not directly to the mass spectrometer; for instance, they do not handle pre-MS sample processing information and protein interaction data.

### Constructing protein-protein interaction network for PARP-1

To illustrate the use of PARPs-DB for discovery of protein-protein interactions, we describe here an experiment for PARP-1 co-immunoprecipitation with interacting proteins. This co-immunoprecipitation is part of experiments aiming at identifying PARP-1 interactors which will be published elsewhere (Ethier et al., manuscript in preparation). Construction of this interaction network involved three bioinformatics steps and the predicted interactions were then verified using standard biochemical techniques.

#### Step1. Identification of PARP-1 interacting proteins from published experimental studies

**Figure 4:**
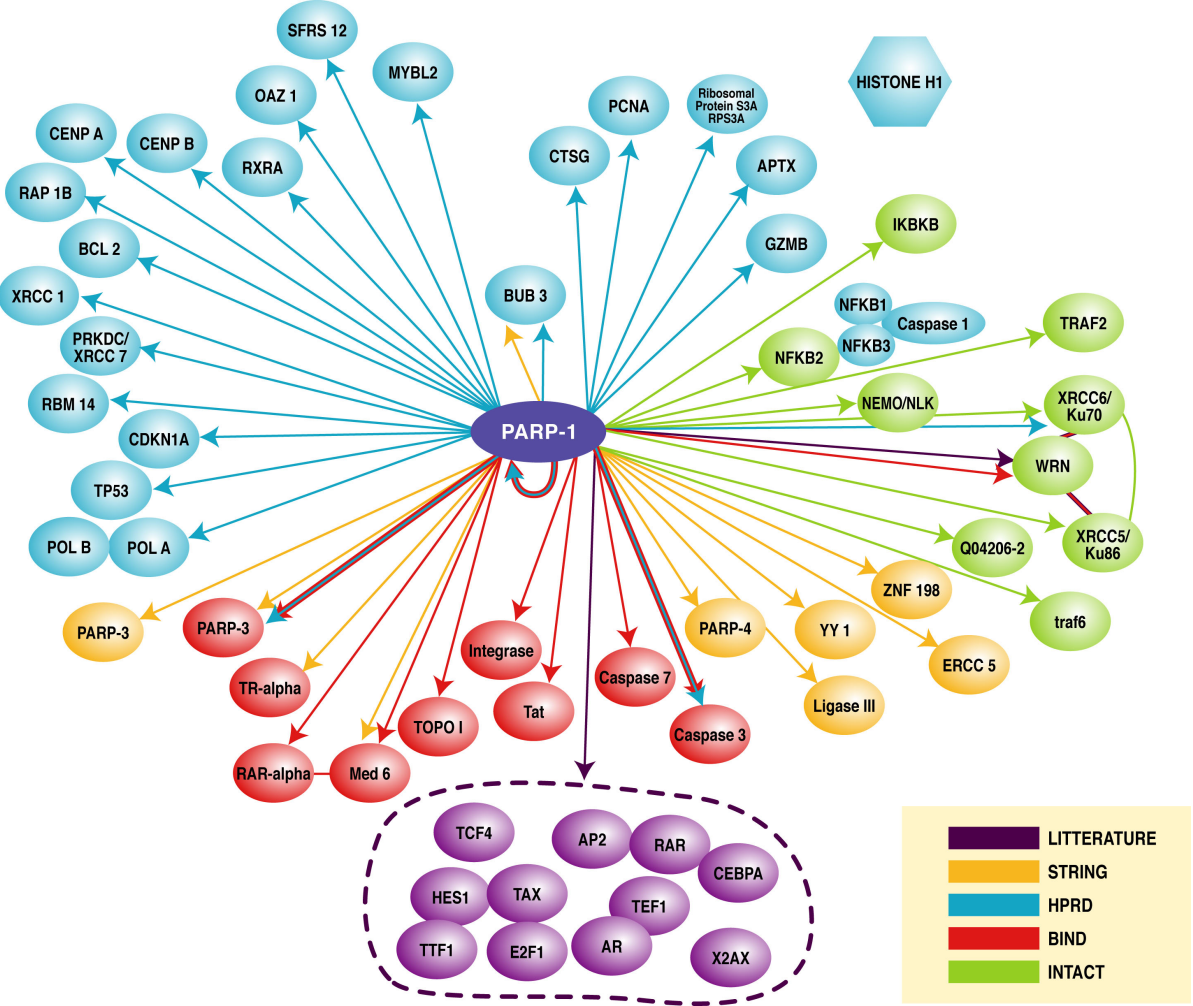
**The biochemical and physical protein interaction network for PARP-1**. This figure is a summary of the results of the protein-protein interaction databases and literature searches for PARP-1 substrates and cooperators. This figure is a hand-drawn representation from the interaction viewer.

The first step in the generation of PARP-1 interaction model is an extensive search of the literature in order to collect published experimental data on PARP-1 interactions. The keyword used in the PARPs-DB search for literature was PARP-1. However, a functional network is not only limited to physical protein-protein interactions but also includes genetic and biochemical interactions (Figure 4).

#### Step2. Establishment of the PARP-1 interaction network by analysis of public databases

The interacting molecules are summarized in Figure 4. Four different comprehensive large-scale yeast protein interaction databases were included in PARPs-DB: BIND, HPRD, INTACT, and STRING. Search within these databases resulted in 52 PARP-1 interactors. BIND, INTACT, HPRD and STRING all have extensive collections of human protein-protein interactions, although the former three databases are primarily used to extract, but not predict, protein-protein interaction data from literature. The database STRING ('Search Tool for the Retrieval of Interacting Genes/Proteins') aims to collect, predict and unify most types of protein-protein associations, including direct and indirect associations. In order to cover organisms not yet addressed experimentally, STRING runs a set of prediction algorithms, and transfers known interactions from model organisms to other species based on predicted orthology of the respective proteins.

One important point in the analysis of data from public protein-protein interaction databases is the quality of the results. Indeed, Deng et al compared the data from all the large-scale yeast interactions screens present in the public protein interaction databases. They developed a maximum likelihood estimation method to access the reliability of the interaction data, and found that the Uetzdata were more reliable than the Ito[8] data, and that the Gavin data were more reliable than the Ho data. Therefore, a cautious use of public databases is indicated. In addition, they suggested that the MS-based analysis of protein complexes performed better in function predictions than the two-hybrid data, thus validating the theory that each component of a complex can be assigned a function based on that of the whole complex. It is clear that yeast two-hybrid and MS-based techniques have both independently made significant impacts on our understanding of the interactome. However, each technique has specific drawbacks that limit the information provided if used alone.

#### Step3. Selection of Protein-Protein Interaction by Gene Ontology Accession

**Figure 5:**
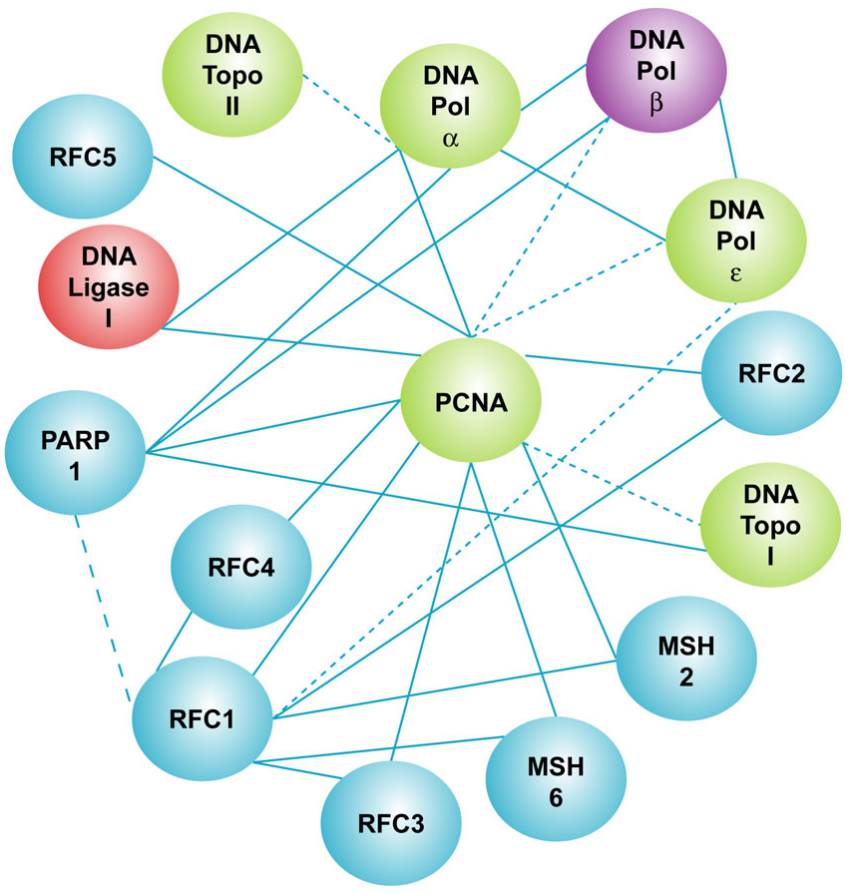
**PARP-1 protein interactions involved in DNA replication**. *In silico *prediction from the protein-protein interaction databases of proteins with a specific function in the replication machinery. To provide maximal coverage of the potential interactome, results from four databases were compiled. These comprehensive database analyses revealed 52 proteins as potential interaction candidates with PARP-1(figure 4). These candidate proteins were further prioritized using searches based on two Gene Ontology keywords (DNA and replication), and 13 proteins were selected on the basis of their unique molecular functions. Further literature reviews identified the proteins in the DNA replication complex, Replication Factor Complex (RFC) complex. Proliferating Cell Nuclear Antigen (PCNA) has been shown to interact biochemically and/or genetically with PARP-1. In this way the potential interactions between PARP-1 and RFC1, 2, 3, 4, 5 was discovered. The solid lines represent direct protein interactions, and the dashed lines represent proteins found in a protein complex. The colour code is described in figure 4.

The next step was to group protein-protein interactions by molecular function via the Gene Ontology [27] controlled vocabulary included in PARPs-DB. Because intracellular events may be compartmentalized to unique intracellular location, to provide additional specificity for target selection we also included a spatial component to further refine the construction of the model. Therefore, we further prioritized our target selection by using two keywords (DNA replication and nucleus). We chose these two keywords to illustrate our approach but PARP-1 is involved in many other cellular processes. After filtering according to function, six out of the 52 initial proteins interactors remained in the networks: PCNA, topoisomerase I and II, DNA ligase I, DNA Pol α and β. An extensive literature search about these proteins in the context of replication and data mining helped to construct a human PARP-1-protein interaction map in PARPs-DB. We found 13 proteins and one complex that interact with PARP-1 in the complex machinery replication. Of the 13 interacting proteins, PCNA, topoisomerase I, DNA ligase I, DNA Pol α and β have been previously reported to interact with PARP-1. Topo2, MSH2, RFC1, RFC2, RFC3, RFC4, RFC5 have been reported to be related to DNA replication. This analysis raises the possibility that PARP-1 may regulate these complexes as a whole rather than regulate one or more the individual components. Of all the potential candidates, we propose that PARP-1 could interact with other proteins in RFC (RFC1 to RFC5) in the context of machinery replication (Figure 5).

#### Demonstration of biochemical interactions between PARP-1 and RFC1

Verifying the interactions from molecules identified *in silico *is vital to provide a confident interaction network useful for further study. With the exception of PCNA, which has been characterized, the prioritized candidates were next tested experimentally to confirm the predicted protein-protein interactions. This was carried out using co-immunoprecipitation of PARP-1 followed by mass spectrometry to identify the interacting proteins.

Scaffold (version Scaffold-01_03_02) was used to group and validate MS/MS based peptide and protein identifications from Sequest, Mascot and X!Tandem. This software is based on the PeptideProphet algorithm which provides an empirical statistical model which estimates the accuracy of peptide identifications made by database search engines. For each tandem mass spectrum, PeptideProphet determines the probability that the spectrum is correctly assigned to a peptide. Scaffold system was used to group the assigned peptides according to corresponding protein and to compute probability of a correct protein assignment for each protein. Peptide identifications with Scaffold software were accepted if they could be established at greater than 80.0% probability as specified by the PeptideProphet algorithm. For the co-immunoprecipitation eluate, protein identifications were accepted if they could be established at greater than 95% probability and contained at least 2 identified peptides. Table 1 lists all the RFC proteins identified with a minimum probability of 95%. It should be noted that the maximal protein probability is limited to 95% in Scaffold. This was set to take into account the light risk that a peptide spectrum match (PSM) is incorrect even if the theoretical and experimental spectra are very similar (e.g: correct peptide absent from the search database).

**Table 1:** List of peptides of RFC-1-5. List of peptides of RFC1-5 subunits identified in an immunoprecipitation of PARP-1 from a HeLa cell lysate by LC MS/MS to the experiment number assigned by the PARPs-DB. This table summarizes the identification from 5 independent immunoprecipitation experiments.

IPI	SwissProt	Protein identification	Gene name	Peptide probability
IPI00375358	P35251	Splice Isoform 1 of Activator 1 140 kDa subunit	RFC1	
		AALLSGPPGVGK		95,00%
		AIVAESLNNTSIK		95,00%
				
IPI00017412	P35250	Splice Isoform 1 of Activator 1 40 kDa subunit	RFC2	
		DAMLELNASNDR		82,70%
		EGNVPNIIIAGPPGTGK		95,00%
		IIEPIQSR		94,10%
		LNEIVGNEDTVSR		95,00%
		LTDAQILTR		95,00%
				
IPI00031521	P40938	Activator 1 38 kDa subunit	RFC3	
		ETANAIVSQQTPQR		95,00%
		KFMEDGLEGMMF		95,00%
		LILCCNSTSK		95,00%
		TVAQSQQLETNSQR		95,00%
		VVLLTEVDKLTK		95,00%
				
IPI00017381	P35249	Activator 1 37 kDa subunit	RFC4	
		AITFLQSATR		95,00%
		ELFGPELFR		95,00%
		GTSISTKPPLTK		95,00%
		IIEPLTSR		91,40%
		ISDEGIAYLVK		95,00%
		LRVLELNASDER		95,00%
		NFAQLTVSGSR		95,00%
		VITDIAGVIPAEK		95,00%
		VKNFAQLTVSGSR		95,00%
		VLELNASDER		95,00%
				
IPI00031514	P40937	Activator 1 36 kDa subunit	RFC5	
		ALNILQSTNMAFGK		95,00%
		FCLICNYLSK		95,00%
		FGPLTPELMVPR		83,00%
		GPILSFASTR		95,00%
		MADIEYR		92,40%
		TSTILACAK		95,00%

The co-immunoprecipitation assay with RFC1 antibody performed in HeLa cells suggests that Replication Factor C subunit (RFC1, 2, 3, 4, 5) (Table 1 and Table 2) formed a complex with endogenous PARP-1. The protein sequence coverage of PARP-1 in this study is 60%.

**Table 2:** List of proteins found. List of proteins found with Sequest and X!Tandem from the PARP-1 immunoprecipitation assay and validated with Scaffold software. Biological sample name refers to the experiment number assigned by the PARPs-DB. This table summarizes the identification from 5 independent immunoprecipitation experiments.

Biological sample name	International Protein Index	SwissProt/Uniprot accession no.	Protein identification	Protein identification probability	Gene name
IP111	IPI00375358	P35251	Splice Isoform 1 of Activator 1 140 kDa subunit	99.8%	RFC1
IP111	IPI00017412	P35250	Splice Isoform 1 of Activator 1 40 kDa subunit	92.9%	RFC2
IP577	IPI00017412	P35250	Splice Isoform 1 of Activator 1 40 kDa subunit	100.0%	RFC2
IP597	IPI00017412	P35250	Splice Isoform 1 of Activator 1 40 kDa subunit	100.0%	RFC3
IP230	IPI00031521	P40938	Similar to human replication factor C (activator 1) 3, 38 kDa	87.7%	RFC3
IP372	IPI00031521	P40938	Similar to human replication factor C (activator 1) 3, 38 kDa	99.8%	RFC3
IP577	IPI00031521	P40938	Similar to human replication factor C (activator 1) 3, 38 kDa	100.0%	RFC3
IP597	IPI00031521	P40938	Similar to human replication factor C (activator 1) 3, 38 kDa	100.0%	RFC3
IP111	IPI00017381	P35249	Activator 1 37 kDa subunit	92.9%	RFC4
IP577	IPI00017381	P35249	Activator 1 37 kDa subunit	100.0%	RFC4
IP597	IPI00017381	P35249	Activator 1 37 kDa subunit	100.0%	RFC4
IP577	IPI00031514	P40937	Activator 1 36 kDa subunit	100.0%	RFC5
IP597	IPI00031514	P40937	Activator 1 36 kDa subunit	100.0%	RFC5
**IP111**	**IPI00449049**	P09874	**Poly [ADP-ribose] polymerase 1**	**100.0%**	**PAPR-1**
**IP230**	**IPI00449049**	P09874	**Poly [ADP-ribose] polymerase 1**	**100.0%**	**PARP-1**
**IP372**	**IPI00449049**	P09874	**Poly [ADP-ribose] polymerase 1**	**100.0%**	**PARP-1**
**IP577**	**IPI00449049**	P09874	**Poly [ADP-ribose] polymerase 1**	**100.0%**	**PARP-1**
**IP597**	**IPI00449049**	P09874	**Poly [ADP-ribose] polymerase 1**	**100.0%**	**PAPR-1**

**Figure 6:**
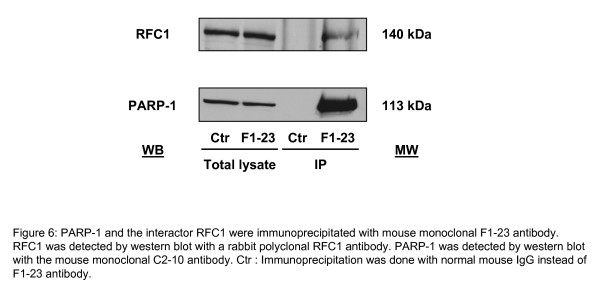
**PARP-1 and the interactor RFC1 were immunoprecipitated with mouse monoclonal F1-23 antibody. RFC1 was detected by western blot with a rabbit polyclonal RFC1 antibody**. PARP-1 was detected by western blot with the mouse monoclonal C2-10 antibody. Ctr : Immunoprecipitation was done with normal mouse IgG instead of F1-23 antibody.

RFC-2, 3, 4 and 5 were each identified with a minimum probability of 95% and by more than 4 peptides with a minimum probability of 95%. The confidence of the identification of RFC-2, 3, 4 and 5 is thus very high and, moreover, we have identified this RFC complex in several co-immunoprecipitates. The case of RFC-1 is different as it was identified by only two peptides of probabilities of 95% and identified in only one co-immunoprecipitate. For this reason, this potential interaction was confirmed by western blot analysis of complexes immunopurified with mouse monoclonal F1-23 antibody (Figure 6). RFC-1 was detected by western blot analysis with rabbit polyclonal RFC1 antibody. As expected, RFC-1 was pulled-down by PARP-1. Although substantially more work is required to determine whether RFC complex interacts directly with PARP-1 or via other proteins such as PCNA, DNA polymerase or through interaction with poly(ADP-ribose), the findings reported here suggest that the PARPs-DB may be useful for finding interacting proteins.

## Conclusion

The work presented here has demonstrated how bioinformatics can supplement conventional biological investigation. The PARPs-DB enables storage, annotation and representation of data generated by molecular biology. Moreover this system has identified a previously unknown protein interaction of PARP-1. The PARPs database allows the effective description of proteomics experiments and analysis of protein-protein interactions.

Because the PARPs database was developed to facilitate data sharing and exchange, it includes the latest standard format to allow sharing of experimental design and results with the scientific community. We have incorporated tools allowing the extraction of protein-protein interactions from the HPRD, DIP and BIND public databases, literature and other sources of information. Reports for peptide and protein analyses are output. These provide comparison reports from multiple or concatenated experiments, thereby significantly increasing the confidence in peptide and protein identifications.

The biochemical data between PARP-1 and RFC complex confirmed the interaction reported earlier. However, substantially more work is required to delineate the specificity and the structural interaction with respect to the regulation of their cellular function between PARP-1 and RFC complex. It is anticipated that the building of such an integrated platform, which can be constantly up-graded, could provide a predictive understanding of a novel gene's function in its biological context. A key design element of PARPs database is the ability to add tools or module that plug into and use the core systems. The PARPs-DB will be expanded as needed in order to make the analyses more efficient.

## Availability and requirements

Project name: PARPs-DB

Project home page: 

Operating system(s) : Unix, Linux, Oracle and MySQL;

Programming Language: Perl, JAVA, SQL;

Licence: GNU GPL;

PARPs-DB is distributed under the GNU GPL licence and available from the website 

## Abbreviations

SQL: Standard Query Language; NCBI: National Center for Biotechnology Information; MS: Mass spectrometry; PARP: Poly(ADP-Ribose) Polymerase; ISB: Institute for Systems Biology; EBI: European Bioinformatics Institute; LDAP: Lightweight Directory Access Protocol; IPI: International Protein Index; HPRD: Human Protein Reference Database; BIND: Biology Interaction Database.

## Authors' contributions

AD implemented, designed the database for the mass spectrometry. MR, CE and APC performed molecular biology approach and helped revise the manuscript. GGP participated and supervised the project. All authors read and approved the final manuscript.

## Supplementary Material

Additional file 1 How to install PARPs-DB. This document gives some help to install Oracle and PARPs-DB Click here for file
