# Supplementary material for: PARPs database: A LIMS systems for protein-protein interaction data mining or laboratory information management system
Source: BMC Bioinformatics. 2007 Dec 19;8:483. doi: 10.1186/1471-2105-8-483 (PMC2266781; doi:10.1186/1471-2105-8-483)
Supplement: Additional file 1 — How to install PARPs-DB. This document gives some help to install Oracle and PARPs-DB [file 1471-2105-8-483-S1.doc]

How To:

**HOW TO**

This installation requires the computer to have a web server (IAS), Perl and Oracle installed. The first three sections explain how to install and configure these components. If those are already installed, there will still be some configuration to do, so make sure to go through all the sections before starting PARPs-DB installation.

There are a couple of other things that are done differently for PARPs-DB and will be noted as we go along.

**Perl**

1. Windows
2. Download and install [ActiveState Perl](http://www.activestate.com/).
3. It is recommended to install it in c:\perl if possible as the Perl scripts expect to find the Perl executable there. Otherwise, the first line of each Perl script will need to be changed to point to the correct location of Perl.
4. Install the dbi and dbd Perl modules needed for database communication. See [here](http://dev.mysql.com/doc/mysql/en/ActiveState_Perl.html) for detailed instructions, or type the following commands at the command prompt.

c:\> cd perl\bin

c:\perl\bin> ppm

ppm> install DBI

ppm> install <http://theoryx5.uwinnipeg.ca/ppms/DBD-mysql.ppd>

1. Unix/Linux
2. In general, Perl is already installed with most of the Linux / Unix distributions.

 Install the dbi and dbd Perl modules needed for database communication.

**Oracle**

Installation of Oracle database is not straight-forward. We strongly suggest installing this component with the database administrator. We have prepared guidelines to install Oracle (see How to install Oracle for Windows or Unix systems). For more information, please consult the official Oracle documentation.

**Database creation**

Once Perl, Oracle and IAS have been correctly installed and configured, the PARPs database can be created.

PARPS scripts are available in the SourceForge.net CVS repository. Those can be checked out through anonymous (pserver) CVS using the following instruction set. The module you want to check out must be specified in the *modulename*. When prompted for a password for *anonymous*, simply press the Enter key.

1. We suggest using the tortoise cvs software, which could be downloaded at the following address for Windows operating systems: <http://www.tortoisecvs.org/>.
   1. Install tortoise software
   2. Create a folder on your local disk to download the files (eg. PARPS_DB)
   3. Right-click on PARPS_DB folder
   4. Select CVS checkout
   5. A new window appears, fill it like in figure 2
   6.
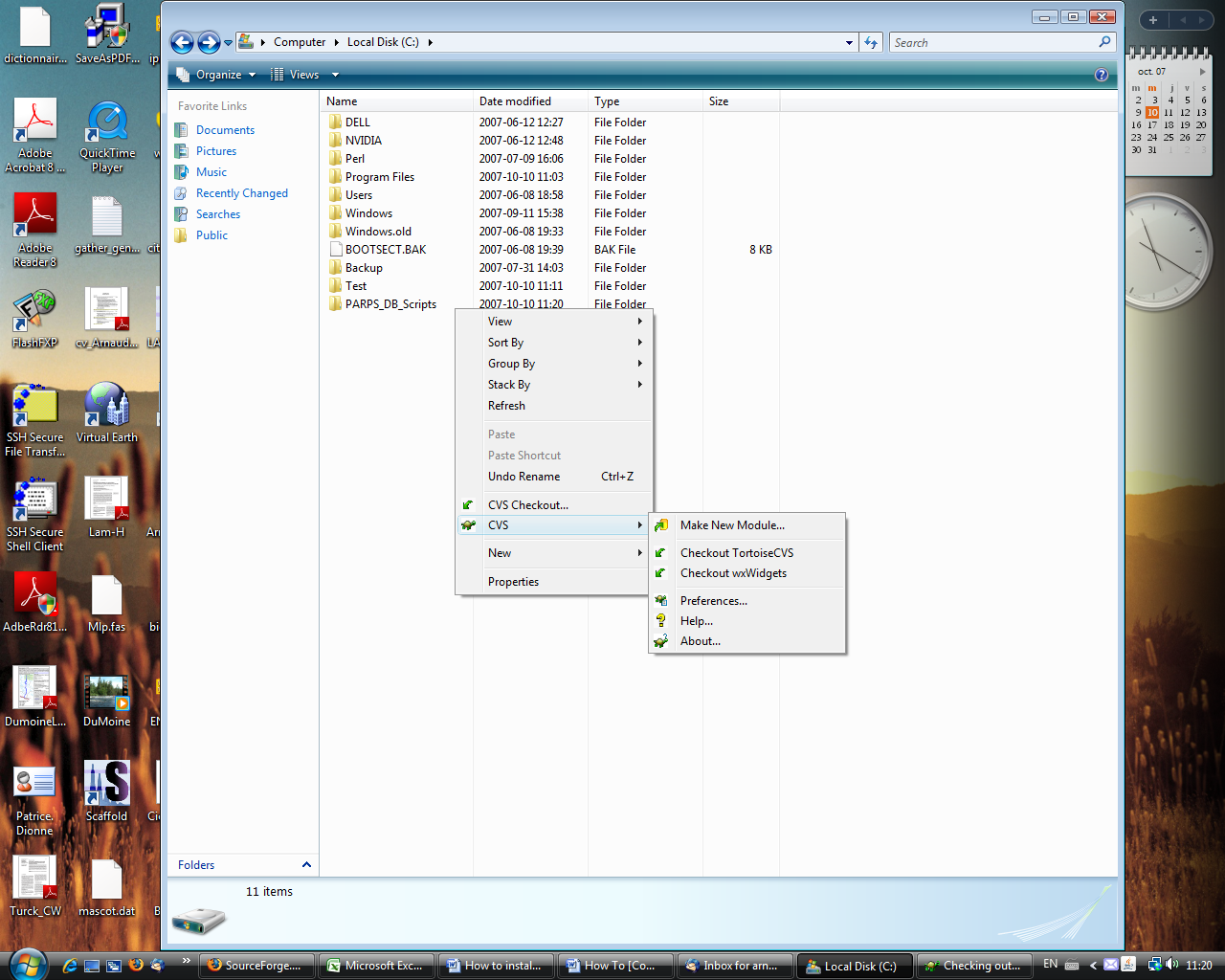
Press ok


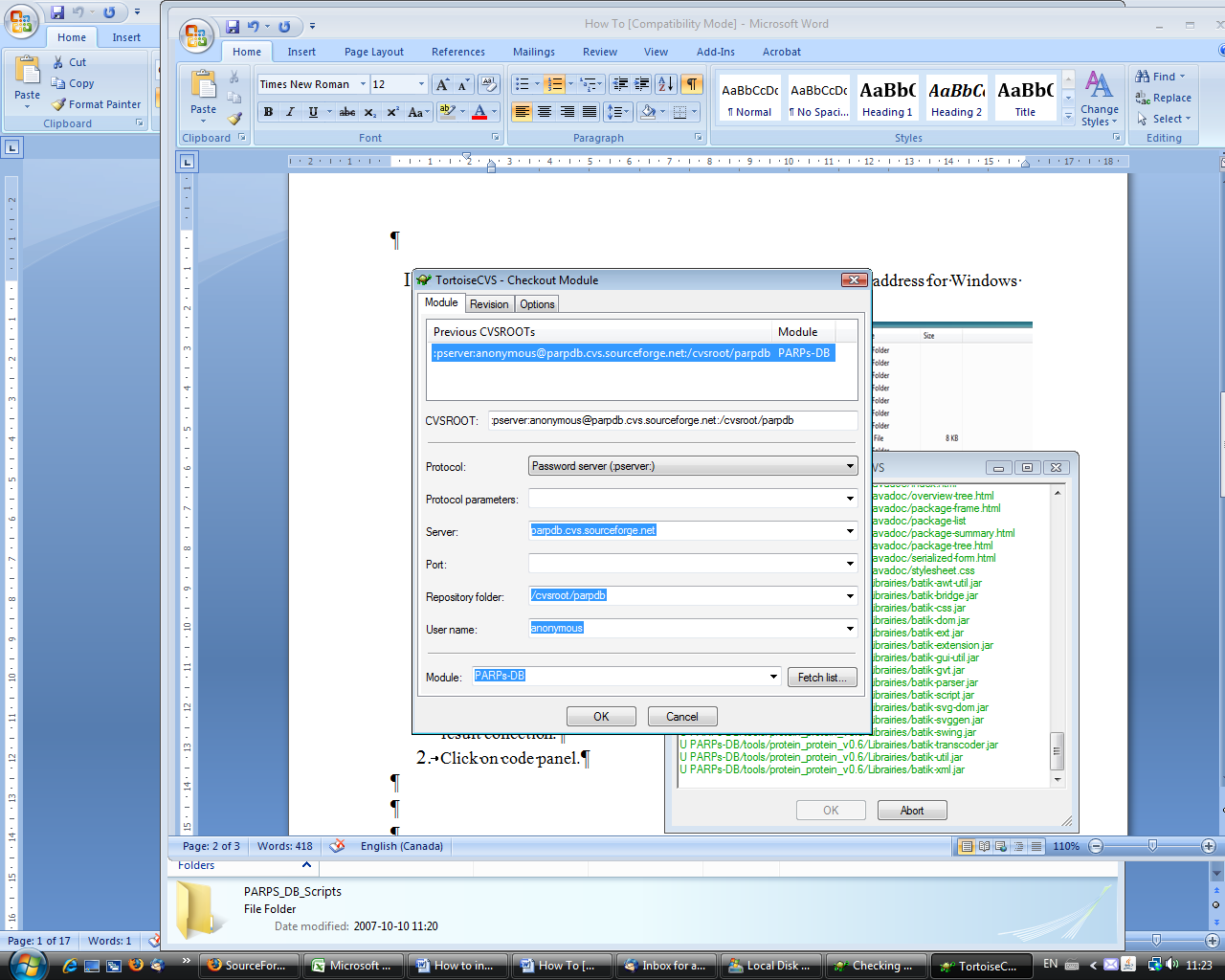


All CVS contents are downloaded into the PARPD_DB folder.

1. With Unix or Linux operating systems, use the following command:

cvs -d:pserver:anonymous@parpdb.cvs.sourceforge.net:/cvsroot/parpdb login

1. All the scripts for the PARPS-DB systems should now be downloaded. All scripts are open source and if you need help, feel free to contact us.
2. Open a command prompt and navigate to the 'scripts_DB' folder in the PARPS_DB_scripts directory.
3. Open a SQL prompt
4. Open the file create_PARPsDB.sql.
5. Copy each instruction of it into the SQL prompt (e.g.

create table project (

project_id INTEGER PRIMARY KEY,

nom_project varchar2(10) );

1. After each successfully executed instruction, you should see :

Query OK, 0 rows affected (0.48 sec)

1. An empty PARPs_DB has been created.
2. The next step is to install PL scripts. Each script must be loaded into the SQL prompt, as in step 5.

Query OK

1. Open the file parp_admin.txt and copy each line into the SQL prompt to grant privileges.

**USE PARPs-DB**

PARPs-Db is now ready to be used. Point your browser to http://servername/index.html. You should see the PARPs-DB start page.

If you get the following error:


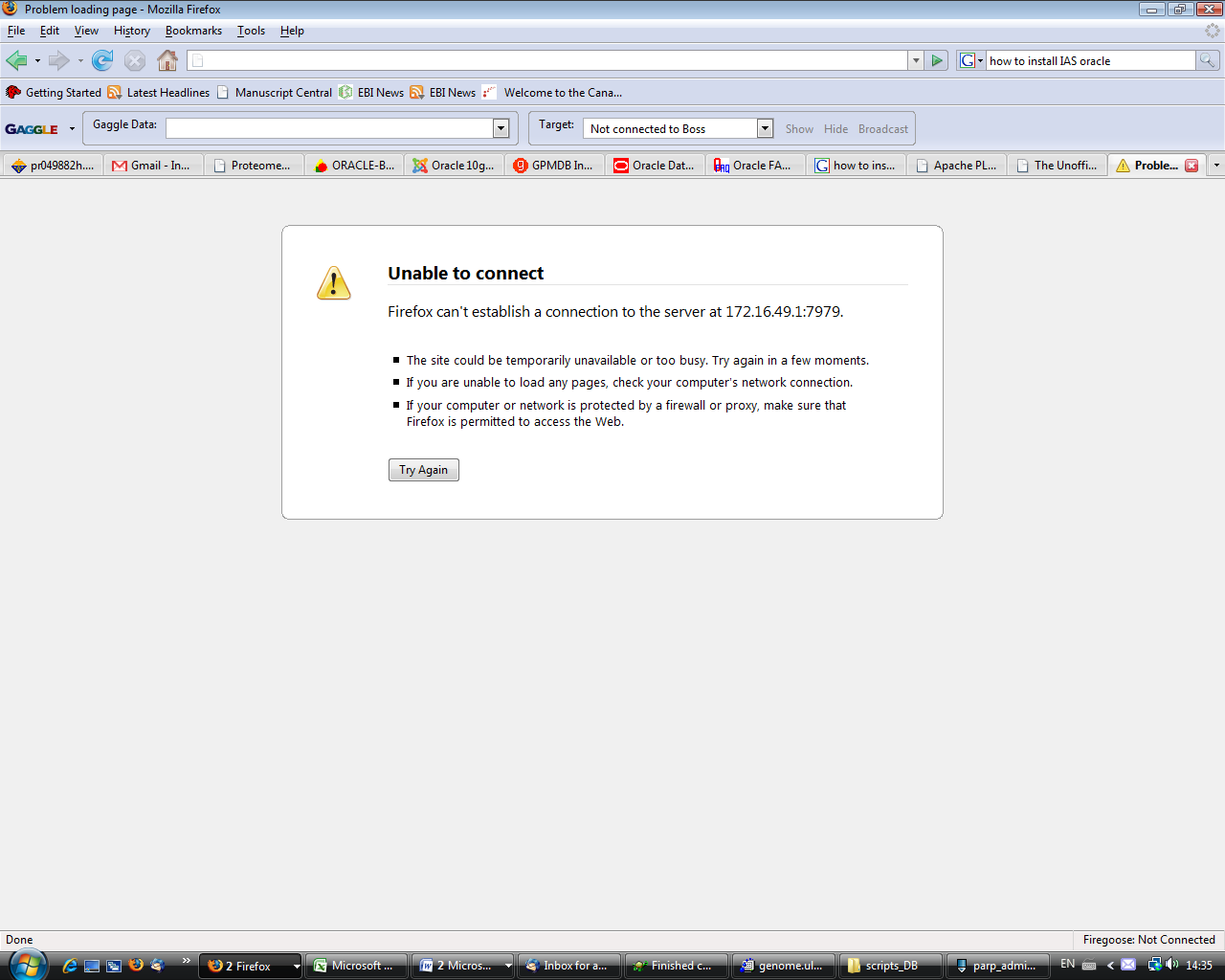


Check the IAS configuration.

**Getting Help**

This set of instructions is not a rigid formula: you may have to adjust the way it is installed based on existing web servers on your computer, or to make it more convenient for you. If you have any problems or questions, email us at arnaud.droit@crchul.ulaval.ca.
